# Art therapy as a comprehensive complementary treatment for Parkinson’s disease

**DOI:** 10.3389/fnhum.2023.1110531

**Published:** 2023-05-12

**Authors:** Tom Ettinger, Marygrace Berberian, Ikuko Acosta, Alberto Cucca, Andrew Feigin, Danilo Genovese, Travis Pollen, Julianne Rieders, Rohita Kilachand, Clara Gomez, Girija Kaimal, Milton Biagioni, Alessandro Di Rocco, Felice M. Ghilardi, John-Ross Rizzo

**Affiliations:** ^1^Steinhardt Graduate Art Therapy Program, New York University, New York, NY, United States; ^2^Department of Neurology, The Marlene and Paolo Fresco Institute for Parkinson’s and Movement Disorders, New York University Grossman School of Medicine, New York, NY, United States; ^3^Doctoral Program in Neural and Cognitive Neurosciences, Department of Life Sciences, University of Trieste, Trieste, Italy; ^4^Department of Exercise Science, Thomas Jefferson University, Philadelphia, PA, United States; ^5^Creative Arts Therapies, Drexel University, Philadelphia, PA, United States; ^6^Zucker School of Medicine at Hofstra Northwell, New York, NY, United States; ^7^Department of Rehabilitation Medicine, New York University Grossman School of Medicine, New York, NY, United States

**Keywords:** art therapy, Parkinson’s disease, neurodegenerative disorder, art making, art based assessment

## Abstract

**Introduction:**

Parkinson’s disease (PD) is the second most prevalent neurodegenerative disease. Complementary and alternative therapies are increasingly utilized to address its complex multisystem symptomatology. Art therapy involves motoric action and visuospatial processing while promoting broad biopsychosocial wellness. The process involves hedonic absorption, which provides an escape from otherwise persistent and cumulative PD symptoms, refreshing internal resources. It involves the expression in nonverbal form of multilayered psychological and somatic phenomena; once these are externalized in a symbolic arts medium, they can be explored, understood, integrated, and reorganized through verbal dialogue, effecting relief and positive change.

**Methods:**

42 participants with mild to moderate PD were treated with 20 sessions of group art therapy. They were assessed before and after therapy with a novel arts-based instrument developed to match the treatment modality for maximum sensitivity. The House-Tree-Person PD Scale (HTP-PDS) assesses motoric and visuospatial processing–core PD symptoms–as well as cognition (thought and logic), affect/mood, motivation, self (including body-image, self-image, and self- efficacy), interpersonal functioning, creativity, and overall level of functioning. It was hypothesized that art therapy will ameliorate core PD symptoms and that this will correlate with improvements in all other variables.

**Results:**

HTP-PDS scores across all symptoms and variables improved significantly, though causality among variables was indeterminate.

**Discussion:**

Art therapy is a clinically efficacious complementary treatment for PD. Further research is warranted to disentangle causal pathways among the aforementioned variables, and additionally, to isolate and examine the multiple, discrete healing mechanisms believed to operate simultaneously in art therapy.

## Introduction

More than 10 million people are affected by Parkinson’s disease (PD) worldwide and each year there are 60,000 newly diagnosed patients with PD in the United States. Based on both prevalence and incidence it is the second most common neurodegenerative disorder after dementia (DeMaagd and Philip, 2015; Parkinson’s Disease Foundation, 2018). Recent research indicates that complementary interventions have the potential to improve symptoms and enhance functioning in PD (Lee and Lim, 2017). The multi-symptom presentation of this progressive neurodegenerative disorder, with no disease-modifying treatment, has encouraged novel complementary interventions to address symptoms and cumulative disability (Kemper, 2019). Mixed methods research, integrating qualitative and quantitative data, has evolved in tandem (Creswell and Clark, 2017). A recently published exploratory study conducted by our team (Cucca et al., 2021) demonstrated overall improvement in motor function and visual-cognitive skills for PD patients after art therapy intervention, as assessed quantitatively through multiple outcomes, including brain connectivity, eye tracking, and clinical and neuropsychological assessments. In what follows we expand on our previous publication by presenting our group PD art therapy intervention and discussing our arts-based, mixed methods assessment.

The pathophysiology of PD is largely related to the progressive loss of dopamine (DA) neurons of the substantia nigra pars compacta (SNc). The basal ganglia are implicated in the execution of internally generated movements, and are involved in procedural learning, habit learning, eye movements, as well as cognition, and emotion. Upon initial diagnosis of PD, almost 40–60% of the nigral dopaminergic neurons are lost (Giguère et al., 2018). In patients with PD, Lewy bodies (intracellular inclusions of abnormal, misfolded protein called alpha-synuclein) are found in the SNc, and are often found widely distributed across the central, peripheral, and enteric nervous systems, thus supporting the well-established notion of a systemic, multifaceted disease (Kon et al., 2020).

The cardinal symptoms of PD include the triad of resting tremor, rigidity, and bradykinesia, the latter defined as slowed or diminished spontaneous and voluntary movements. Additional motor symptoms may include postural instability, balance impairment, dystonia (painful muscle contractions), and dysarthria (slurred or slow speech) (Kalia and Lang, 2015; Cucca et al., 2018). Pharmacological treatment of the disease has predominantly focused on motor dysfunction. However, insofar as motor activity involves sensorimotor feedback loops, the degeneration of perceptual capabilities is an equally important research topic. Difficulties in motor and perceptual functioning, furthermore, are not isolated impairments, rather they negatively influence broad aspects of a PD patient’s biopsychosocial being (van Uem et al., 2016; Cucca et al., 2018).

Parkinson’s disease psychiatric impairment can include obsessive-compulsive disorder, phobias, and severe anxiety over the deterioration of bodily functions (Khatri et al., 2020). Some of these manifestations, like obsessive-compulsive behaviors and perceptual abnormalities, may arise as the result of a complex interplay between the underlying neurodegenerative disease and the exposure to dopaminergic treatments, thus further complicating the pharmacological management of these patients. Additional signs and symptoms of the disease include hypomimia, a reduction of facial expressivity that results in a fixed, mask-like appearance (Gunnery et al., 2016), as well as dysgraphia, defined as the inability to execute fluent handwriting, with deviations in size, duration, and velocity (Thomas et al., 2017). Writing can be evaluated kinematically (through frequency, amplitude, direction and symmetry) to examine abnormalities related to tremors and hypometria associated with PD (De Pandis et al., 2010; Alty et al., 2017).

Regarding affective dysregulation, the prevalence of reactive depression, apathy, anxiety disorders, and mental/physical fatigue in PD all hover around 50% (Friedman et al., 2016; Upneja et al., 2021; and Chikatimalla et al., 2022). When compared with patients burdened by other chronic disabling conditions, and matched for disability severity, PD patients display an increased risk for endogenous mood dysregulation (Almeida et al., 2020). The underlying neural substrates and pathways to affective dysregulation remain largely unknown. The degeneration of mesocortical and mesolimbic dopaminergic neurons may disrupt the serotonergic signaling in the dorsal raphe, thus resulting in a dysfunction of orbitofrontal-basal ganglia-thalamic circuits involved in mood regulation and reward.

Regarding cognition, approximately 30% of newly diagnosed PD patients report subjective cognitive decline, and 20% have mild cognitive impairment (MCI) (Pan et al., 2021). Traditionally, two main patterns of cognitive involvement have been identified in PD. The so-called “anterior” pattern arises as a consequence of the disruption of dopaminergic signaling across frontal and pre-frontal areas, manifesting with a characteristic dysexecutive syndrome affecting the ability to adapt to new contexts, problem-solving, mental speed, and cognitive flexibility. Conversely, the so-called “posterior” pattern is traditionally attributed to the cholinergic involvement of the temporal and parietal cortices, accounting for memory impairment and visuospatial dysfunction. During the course of the disease, psychotic symptoms, including hallucinations, delusions, and atypical thought disorders, can be experienced by up to 40% of patients (Barnes and David, 2001). These symptoms typically occur in the most advanced stages of the disease, and they are particularly frequent in patients with overt dementia.

In the absence of established neuroprotective strategies countering the degeneration of dopaminergic neurons and associated circuitry, pharmacological treatment is mostly limited to managing motor symptomatology (Raza et al., 2019). However, prolonged use of medications often leads to side effects, including dyskinesia, as well as medication intolerance (Rana et al., 2015; Lee and Lim, 2017). For this reason, development of complementary therapies is especially important.

### Neurological findings previously published

Results from our recently published Cucca et al. (2018, 2021) exploratory, open label study conducted with 18 non-demented PD patients with mild to moderate PD yielded multiple clinical improvements following 20 sessions of visual art therapy. Specific improvements were noted in different visuospatial functions, including visuoconstructional abilities as indicated by performance on the Rey Figure test (immediate copy), figure/ground segregation as indicated by the number of errors on the Navon Test, and in visual exploration strategies as assessed by means of an eye tracking procedure (reduction in saccadic path length, with the development of more efficient eye scanning on Benton figure recognition test). Post-treatment Resting State fMRI (rs-fMRI) evidenced significantly increased functional connectivity (FC) in both primary and secondary visual networks, suggesting a functional reorganization of neural networks mobilized by art therapy training. The benefits extended to motor function, including gait profile, as evidenced by changes in UPDRS-III, Timed Up and Go test. Indices of overall quality of life improved as well.

Our past publications were restricted to presenting clinical outcomes as well as neuropsychological tests and brain imaging results. In the present publication, we report on a further aspect of our research program. We developed a visual arts-based assessment instrument specifically criterion-keyed to PD. The rationale is that an assessment instrument that matches the treatment modality will be maximally sensitive to the effects of the treatment. In art therapy, clinicians implicitly follow the same strategy, periodically deploying standardized drawing tests (like the Kinetic Family Drawing test) to get a better sense of how the client is progressing in specific psychological areas of interest. Our test is the House-Tree-Person Parkinson’s Disease Scale (HTP-PDS), described shortly.

### The arts as complementary interventions

The impact of engagement in the arts to support physical and mental health for a range of medical conditions has been widely recognized by both patients and healthcare providers (Crone et al., 2018). Growing evidence details the role of the arts and culture in promoting health and well-being throughout the life course (Lomas, 2016; All-Party Parliamentary Group on Arts, Health and Wellbeing, 2017; Hanna, 2017). Long-term, sustained arts engagement offers prolonged health benefits for older adults including higher life satisfaction and eudaimonic well-being (Tymoszuk et al., 2019). More recently, social prescribing, defined as non-medical interventions to “address wider determinants of health and to help patients improve health behaviors and better manage their conditions,” has increasingly gained support (Drinkwater et al., 2019, p. 1). A recent systematic review of the arts and humanities found music, visual arts, and literature interventions associated with increases in broad measures of “flourishing” (Shim et al., 2021).

Art therapy as a complementary intervention improves multiple domains of functioning. Within the structured psychotherapeutic relationship, art therapy integrates sensorimotor activity with cognition and affect, and it fosters self-esteem, self-awareness, and resiliency (American Art Therapy Association, 2017). Art therapy recruits and integrates myriad neurologic mechanisms that underpin art-making, which involves sensory-motor integration, hand-eye coordination, non-verbal visuospatial processing, memory, abstraction, and so forth (Cavanagh, 2005; Johnson, 2009; Hass-Cohen and Findlay, 2015). Distributed, interdependent networks in the brain are involved in creativity itself, apart from any given medium or modality (De Pisapia et al., 2016). The adaptive nature of creativity, considered an evolutionary mechanism similar to playing, promotes growth, flexibility, and experimentation, or trial action (Schore and Marks-Tarlow, 2018).

Research studies on creative visual expression have documented enhanced motor functioning (Elkis-Abuhoff and Gaydos, 2016, 2018; Tickle-Degnan et al., 2010); reduced cortisol levels (Kaimal et al., 2016), improved mood and self-efficacy (Kaimal and Ray, 2017); decreased distress (Glinzak, 2016); reduced anxiety (Jang et al., 2018); reduced pain (Shella, 2017); increased emotional processing (Canesi et al., 2012; Faust-Socher et al., 2014), improved emotional awareness (Montag et al., 2014), and improvement in overall quality of life especially in the amelioration of depressive symptoms (Bae and Kim, 2018). In a quantitative EEG study, King et al. (2017) documented increased alpha and beta wave activity, associated with a relaxed alert state, following art making, as compared to rote activities. These findings are supported by recent neuroscience research in artistic production evidencing multiple, interconnected brain regions (Benedek et al., 2014; Boccia et al., 2015; Corazza, 2016). Also noteworthy, art-making activates dopaminergic responses, suggesting art therapy has the potential to induce other parts of the brain to compensate for the damages of PD through neuroplasticity (Mirabella, 2015).

Regarding sculpture in visual arts therapy, an exploratory study with PD patients involving 16 sessions of clay-based art therapy reported significant improvements in hand dexterity, self-expression, mood, depression, and quality of life as compared to a non-equivalent control group with maintained routine rehabilitation program (Bae and Kim, 2018). Clay requires visuospatial processing in the three-dimensional world, and it provides a tactile medium with enhanced sensorimotor feedback. Other arts modalities have proven useful in treating PD. Theater training was found to enhance emotional well-being and facilitate cognitive rehabilitation in patients with PD, compared to a control condition of conventional physiotherapy (Modugno et al., 2010; Mirabella et al., 2017). Theater based therapy, like art therapy, involves absorption, fantasy, and play. Theater, akin to drama therapy and certain somatic therapies, advantageously adds the real world factors of full body movement and interpersonal interaction, both of which transfer to daily functioning outside the theater setting (Mirabella et al., 2017). Modugno et al. (2010) found theater to be broadly efficacious as measured by improvements in diverse clinical scales [Unified Parkinson’s Disease Rating Scale (UPDRS), Schwab and England Scale, Parkinson’s Disease Quality of Life (PDQ39) Scale, Epworth Sleepiness Scale (ESS), and Hamilton Depression Rating Scale (HDRS)].

There is also substantial research on art viewing for neurologically impaired persons. Viewing art involves some of the same processes as making art, such as absorption, fantasy, and reward. Viewing art can be a challenging process that unfolds over time, with aesthetic attunement and interpretation involving complex visual problem-solving skills. Viewing art involves empathy, an ideational re-creation of the artists’ intentions and actions (irrespective of accuracy) (Ettinger, 1999). Viewing great art imparts a sense of connectedness with culture at large, which is a strong antidote to the sense of isolation imposed by neurological illness. Many, if not most major museums have recently developed community involvement programs to tap into the mental health benefits of the arts, especially for the elderly (Hartman, 2022). As is widely reported by participants, and increasingly demonstrated through research, art viewing imparts general emotional well-being, and strengthens cognitive skills that transfer to the real world. For example, it begets improvement in working memory and memory consolidation on non-art tasks (Ciccarelli et al., 2019).

Figueroa (2022) concludes that art therapy offers an optimally flexible, ecologically valid and holistic paradigm for rehabilitation, enhancing a broad array of biopsychosocial skills to promote effective coping in the face of a devastating and incurable disease. Still art therapy, and complementary interventions generally, have suffered from a paucity of properly designed and sufficiently powered research. This is due to the recency of these approaches and to the inherent complexities of researching complementary interventions. These approaches involve multiple interconnected phenomena such as esthetic reward, motor training, environmental enrichment, socialization, and visuospatial training. It is therefore challenging to isolate and measure variables, and to disentangle causal pathways, and yet more complicated to identify underlying neural substrates. Further, despite a growing number of medical practitioners affirming the benefits of artistic engagement, complementary approaches are still rarely incorporated into standards of care for these clinical populations.

### Art-based assessment

Art-based assessment is presently a unique and valuable tool in understanding complex illness. The profession of art therapy has dual roots in the systematic structural analysis of drawings (Buck, 1948; Arnheim, 1954; Hammer, 1958; Burns and Kaufman, 1970; DiLeo, 1970) and in psychoanalytic speculations about the unconscious, personality, and creative artistry. These converged to catalyze the development of projective tests for diagnostic purposes. Historically notable tests include the scribble test (Cane, 1931), the Kinetic Family Drawing (Burns and Kaufman, 1970), and the MARI Card Test (Kellogg, 1980).

Traditionally, standardized and quantitative assessment methods have sought to remove subjectivity, prioritizing ease of measurement over human complexity and subjectivity. Art-based analysis, conversely, seeks to uncover each person’s complexity and subjectivity. To this end, arts assessment values equally the overtly recognizable, culturally shared, depicted content and the more covert, personal meanings connoted and encoded in the formal elements of the image (such as color and texture) (Rubin, 2009). The FEATS (Formal Elements Art Therapy Scale) is the pre-eminent validated instrument that analyzes Formal Elements (Gantt, 2015).

The field of clinical psychology emerged from the same psychoanalytic roots in parallel with art therapy. The psychologist’s test battery usually includes five to eight tests, mixing objective and projective formats as well as response modalities (verbal, visual, and motoric). Psychologists in the psychodynamic tradition also utilize the House Tree Person (H-T-P) test, and also attend to the projective aspects of both content and form. Psychologists utilize a wide array of projective tests consistent with the H-T-P. For example, the Rorschach test is scored according to the dual domains of content and formal variables. The latter are termed the “determinants” of percepts, and they include outline, color, movement, shading, and achromatic color (black/white used as colors).

In art therapy, H-T-P interpretation is contextualized in ongoing clinical work, which includes verbal dialogue; consequently H-T-P interpretation is oriented and refined by copious extrinsic information. Similarly, in psychological assessment, H-T-P interpretation is contextualized among multiple other tests in the test battery. These typically include the Bender Gestalt test, which entails copying geometric figures, in sight and from memory. The WAIS-IV IQ test includes ten subtests that are equally visual and verbal, with several requiring motoric responses. The scoring yields four Indexes: Perceptual Reasoning, Processing Speed, Working Memory and Comprehension. Comparing subscales, by empirically derived formulas, yields substantial information in the case of neurodegenerative disorders, even disentangling pre-morbid from disease induced levels of functioning. Also, a psychologist adds patient-specific tests to the battery, and for PD patients, the H-T-P test is further contextualized among neuropsychological tests. Thus as with art therapy, the psychologist’s H-T-P interpretation is oriented and refined by a broad array of extrinsic information. Clinical training in both fields is predominantly guided by expert supervision in internships, which substantially augments what a trainee can learn from the test manuals and the research literature.

The H-T-P is an ideal projective drawing test because it includes a human figure, an animate tree with symbolic properties suggesting an unconscious self-image (How do the roots meet the earth—is the tree well-grounded or unstable or floating or clinging? Do the branches reach out to the environment or fold inward protected by a circular canopy? Is it enlivened or barely alive?), and a house that includes personal and interpersonal aspects (is there light, heat, and an inhabitant(s), or is it vacant? Is it accessible to others, with windows and a walkable path, or is it shut off from the world?). There are variations on administration, but ideally the three items are drawn on a single page. There are several excellent projective tests that are however, geared toward a specific population (such as the Kinetic Family Drawing Test) or a more circumscribed set of psychological issues (such as the Person Picking an Apple from a Tree drawing test, which is ideal for assessing attachment style).

Research on the H-T-P and PD is scant but informative. Kulkarni et al. (2013), for example, noted PD artists have pervasive difficulties connecting lines when drawing a house with windows and a door. PD artists proved unable to connect the door to the bottom of the house, producing a floating door; however, it remains unclear if this is predominantly a sensorimotor or a visuospatial difficulty.

In summary, art-based assessment is ideally positioned to capture a broad array of ecologically valid data that eludes more traditional assessment modalities, albeit the complexity of art carries inherent liabilities regarding reliability and validity.

## Materials and methods

### Study design

The “ExplorArtPD” study is an original, multidisciplinary research initiative, carried out at the Marlene and Paolo Fresco Institute for Parkinson’s and Movement Disorders, NYU Langone Health, New York City, USA (clinicalTrial.gov; identifier: NCT03178786). This research explored the impact of 20 sessions of art therapy intervention on a group of PD participants, focusing on motor symptoms, visuospatial exploration, visuomotor integration, neuropsychological, and emotional spheres, and quality of daily living. The findings from clinical tests, neuropsychological inventories, ocular physiology, and MRI brain imaging were published in Cucca et al. (2018, 2021). This research is a prospective, open-label trial that assesses the rehabilitative potential of art therapy for PD. Participants were assessed before the first session of art therapy and within 2 weeks from the last session. We refer the reader to the previous publications for the exact study design and primary neurological outcomes of the ExplorArtPD study. The present paper will discuss the results obtained with a further, unpublished assessment instrument, the House-Tree-Person PD Scale (HTP-PDS).

### Participants

The PD group was recruited among a cohort of patients enrolled in the ExplorARTPD Study with mild to moderate PD, per the United Kingdom Parkinson’s Disease Society Brain Bank criteria (Hoehn and Yahr stage 2–3, *M* = 2.3, SD = 0.5; duration in years *M* = 6.2, SD = 4.6). Hoehn and Yahr stage 2 reflects disease areas of bilateral or midline involvement without impairment of balance. Stage 3 reflects bilateral disease progression with mild to moderate disability and impaired postural reflexes; patients are physically independent in daily living. From September 2017 to January 2020, five PD cohorts (8–10 participants in each group, *n* = 42), were enrolled. Participant age ranged from 54 to 90 (*M* = 68.4 years; SD = 6.0); Female/Male ratio = 30:12. Exclusion criteria are severe motor fluctuations, bothersome dyskinesia, severe fluctuations in attention, alertness or cognition, psychotic diagnoses, and marked inability to manipulate art materials. Baseline measures showed equivalence between the groups demographically including age, gender, years of education, general cognitive abilities, and hand dominance.

### Study objectives and hypotheses

The ExplorARTPD project examined whether art therapy can ameliorate the core PD symptoms of motor control and visuospatial dysfunction, and whether improvements here will beget positive changes in other functional and experiential domains. It is hypothesized that art therapy will produce positive effects on motor and visuospatial functioning, and more generally, on cognition, mood, motivation, self-image, self-efficacy, interpersonal functioning, creativity, and global functioning, as measured by our arts-based H-T-P assessment.

We will further examine whether the art therapy assessment results directly correlate with the neurological assessment results reported by Cucca et al. (2018, 2021). Art therapy is likely to involve learning and practicing of peculiar functional domains presumably mediated by complex neural substrates, which are not necessarily reflected by changes in traditional clinical and neuropsychological outcomes. This is especially likely for PD patients, insofar as the acquired and evolving sensorimotor skills are relatively spared by the neurodegenerative process of PD, as the latter is known to mainly affect the planning and execution of semiautomated, internally generated motor tasks.

A further reason to be cautious about expecting correlations with neuropsychological test results is that aspects of our arts assessment, such as the Creativity scale, are scored irrespective of pain and dysfunction. Creativity is a resource, like resiliency, and it reflects the capacity for symbolic compensation, reparation and rejuvenation. Paradoxically creativity can be piqued, activated, and enhanced by increased pain and dysfunction. Other aspects of our arts assessment tap into the interaction of these negative aspects with self-regulation and self-control skills. It is not clear how these dynamics might correlate with traditional PD neuropsychological measures.

### Art intervention/treatment

The experimental protocol of the project was previously published (Cucca et al., 2018). Intervention consists of 20 structured group art therapy sessions (twice weekly, 90-min sessions for 10 consecutive weeks) involving varied media. A total number of 364 sessions of art therapy were administered, with overall 91.5% adherence.

### Art assessment: the HTP-PDS

The House-Tree-Person (H-T-P) is a longstanding assessment instrument in the dual domains of art therapy (Hammer, 1958) and psychological assessment (Ettinger, 1999). The House-Tree-Person-Parkinson’s Disease Scale (HTP-PDS), developed by Ettinger et al. (2018), is a novel art-based scale that combines qualitative and quantitative methods for use in nomothetic research. It was developed (1) based on the HTP literature, (2) based on the PD literature, (3) based on clinical familiarity with the HTP and with PD patients, and (4) based on sorting notecards generated while studying the set of PD patient drawings to be assessed. (5) The Clinical Scales largely correspond to the paragraph sequence in clinical psychological test reports on PD patients. Additionally, (6) test construction principles were strictly adhered to (American Psychological Association, APA Task Force on Psychological Assessment and Evaluation Guidelines, 2020). The Scale therefore has threshold content and face validity. Because the HTP-PDS is criterion keyed to PD drawings, it has fine distinctions in areas such as line quality, an indicator of motor control, and visuospatial processing. As noted above, participant criteria excludes psychosis, however, the scoring system is expanded to assess more severe thought and mood disorder variables, with the intention that the assessment be valid for all PD patients irrespective of severity or comorbidities.

The assessment involves drawing a house, tree and person on a single 18 x 24 sheet of paper with crayons, pencils and an eraser. The instructions impose no structure beyond this. There is no time limit.

The HTP-PDS scoring involves two phases. The first phase considers Observable Pictorial Variables across eight scoring categories. The rater scores the drawings using a five-point Likert scale, with endpoints titled impaired and intact. The first five categories consider pictorial form (as opposed to content):

1. line quality (this captures motoric aspects, such as firm/weak, continuous/broken-frozen, smooth/wobbly, and ability to execute fine line placements when intended),
2. visual/spatial aspects (including part-whole or local-global processing, 3D construction accuracy, attention to and articulation of fine details, and integrational and organizational aspects),
3. cognition: logic/thought (based on the Rorschach Special Scores, this tallies visible condensations, incongruous combinations, illogical sequences, and related illogical phenomena),
4. energy, and
5. color.

The next three Scale categories consider pictorial Content (as opposed to Form):

6. person (as a self-projection, the image conveys body-image and internal psychological aspects such as self-efficacy, emotion, and motivation; one may also discern interpersonal aspects, because the person is interacting with the environment and with the implied viewer/audience),
7. tree (a less conscious self-symbol), and
8. house (a less conscious symbol of internal and interpersonal resources).

In the second phase, the aforementioned ratings are mapped onto Clinical Scales that capture phenomena of interest. As implied above, the first pictorial variable, line quality, is mapped onto the first scale:

1. motor control.

The next two clinical scales are titled:

2. visual/spatial processing and
3. cognition: logic/thought.

The latter two scales mirror the corresponding first phase pictorial variables that are similarly named. There are however a few exceptions. For example, the use of color (pictorial variable 5) that is arbitrary, odd, or incorrect indicates possible cognitive disorder, so it is mapped onto clinical Scale 3 (cognition: logic/thought).

Here are the remaining clinical scales, based on diverse variables from both Form and Content (house, tree, and person):

4. affect/mood,
5. motivation (note that Energy – pictorial variable 4 – bears upon Motivation),
6. self,
7. interpersonal, and
8. creativity. Creativity is scored independent of accuracy, logic, angst, and dysfunction (so for example, incorrect color can dually be scored as creative). The last scale item is:
9. global assessment of functioning. This is based on the DSM-IV scale that integrates all weaknesses and strengths. Finally, raters described:
10. Observations, in narrative form (such as conflict areas and control mechanisms and resources for self-regulation; these are not tallied quantitatively but they are used to refine scoring generally).

A scoring manual was drafted, intended for use by psychodynamically trained art therapists or clinical psychologists trained on the H-T-P. As with all arts-based assessment instruments, the manual lists criteria, however, it requires that the viewer have substantial background knowledge and experience. For example, the Person body-image criteria is essentially a checklist of pictorial aspects that the rater is to consider and weigh.^1^

### HTP-PDS administration

The HTP-PDS was administered individually at baseline and within the week following the twenty art therapy sessions for the majority of study participants (*n* = 37). Five of the participants were unable to participate in the assessment due to personal logistics. For patients experiencing motor fluctuations, assessments were administered in the ON-state to minimize fatigue and physical discomfort.

### Data scoring and statistical analysis

Three experienced clinicians performed the assessments and scored the protocols. The raters were blind to condition but not to the research hypotheses. Furthermore, all pre/post-drawings were coupled as pairs and scored together. Discrepancies among raters were resolved by discussion or by averaging ratings. Regarding interrater reliability, the clinicians trained three recent art therapist graduates on the scoring manual, and they scored a subset of protocols double blind. Agreement was defined as two raters agreeing that one drawing was superior to another when assessing a pair of pre/post-drawings, for any given scale. Agreement was examined between each pair of the three raters, and it was determined to be acceptably high, with a range from 80 to 87.5% agreement for all nine scales.

Difference scores were calculated for the clinical tests and art assessment categories, and normality of those difference scores was assessed using the Shapiro–Wilk Test and visual inspection of histograms. Central tendencies and dispersions of pre- and post-intervention were calculated as mean ± standard deviation for normally distributed data and median (interquartile range) for non-normally distributed data. Differences between pre- and post-intervention scores were assessed with one-tailed paired *t*-tests for normally distributed data and one-tailed Wilcoxon Signed Rank tests for non-normally distributed data. Effect sizes were also calculated for normally and non-normally distributed data as Cohen’s d and r, respectively. Lastly, a correlation matrix was calculated on the differences scores of the art assessment categories to assess relationships between changes in each category. Pearson’s r and Spearman’s ρ were used for correlations between normally and non-normally distributed data, respectively. Statistical significance for all tests was set to α = 0.05, and all tests were run in IBM SPSS Statistics (Version 27).

## Results

Mean scores by blind raters of the HTP-PDS are displayed in Table 1 and demonstrate positive gains across all scales, with medium to large effect sizes. Based on the correlation matrix for the difference scores, differences in all categories were significantly positively correlated with differences in all other categories (Pearson’s r and Spearman’s ρ between 0.43 and 0.79, *p* ≤ 0.008). For all variables, changes from pre to post were highly correlated. The HTP-PDS intercorrelations confer internal consistency upon the scale and therefore some additional evidence of construct validity. The results indicate that art therapy positively affects PD symptomatology and overall biopsychosocial functioning. Anecdotally, the scale results are supported by self-reports and reports from significant others (elicited in narrative form, not quantified). Also anecdotally, participants had different patterns of motor control dysfunction. The particular dysfunctions tended to improve and ameliorate, whereas unimpaired motor functioning indices only improved from an aesthetics perspective, as would be expected from any art student. The pattern was slightly different for visuospatial processing. All aspects were more homogeneous and tended to improve in tandem—integration, differentiation, and articulation; compositional balance, and sophistication in 3D rendering.

**TABLE 1:** Mean ± standard deviation or median (interquartile range) pre- and post-intervention scores for *N* = 37 participants.

Category	Pre-treatment HTP-PDS [mean ± SD or median (IQR)]	Post-treatment [mean ± SD or median (IQR)]	Difference between pre and post-treatment mean or median scores	*p* (1-tailed)	Effect size
Motor control	3.0 (2.0)	4.0 (1.8)	+1.0	<0.001*	*r* = 0.70
Visual/spatial functioning	3.0 (0.8)	3.5 (1.0)	+0.5	<0.001*	*r* = 0.61
Cognition: logic/thought	4.0 (2.5)	5.0 (1.5)	+1.0	0.011*	*r* = 0.38
Motivation	3.0 ± 1.0	4.1 ± 0.8	+1.1	<0.001*	*d* = 0.93
Emotion	2.5 (1.8)	4.0 (0.8)	+1.5	<0.001*	*r* = 0.69
Self	2.6 ± 0.9	3.5 ± 0.8	+0.9	<0.001*	*d* = 0.73
Interpersonal relatedness	2.6 ± 1.0	3.4 ± 1.0	+0.8	<0.001*	*d* = 0.61
Creativity	3.0 (1.8)	4.0 (1.5)	+1.0	<0.001*	*r* = 0.73
Global assessment of functioning	3.0 (2.0)	4.0 (0.5)	+1.0	<0.001*	*r* = 0.61

SD, standard deviation; IQR, interquartile range. All categories improved significantly from pre- to post-intervention with medium to large effect sizes. *P < 0.05.

Each Clinical Scale is derived from multiple Observable Pictorial Variables. However, we did not perform statistical calculations on the latter. The reason is that we did not require that they be weighed equally when determining the corresponding Clinical Scale scores. By way of example, Motor Control is based on five Line Quality variables. Participants can get a high impairment score based solely on one item, such as inability to execute fine line placement when intended (often seen in window depictions), or pervasive stop-start line work. The artistic differences are likely due to two factors: differing PD symptom expression and differing approaches to pictorial representation (based on internal schema, from earlier arts viewing and practice). The factors are yet to be pinpointed and disentangled.

Similarly, the checklist for rating the Person element of the drawing, considering body-image alone, contains some dozen variables to consider, and remains open-ended for idiographic aspects. While raters are able to agree on whether pre- or post-body-image is superior, it is not feasible to quantify and weigh checklist items. We did, however, make progress in this daunting task, facilitating a more systematic approach in future research.

Art therapy pivots on creativity. The creativity scale—the most elusive of all scales—measures immersion in and mobilization of inner resources. Creativity in general involves the integration of divergent and convergent thinking. In art therapy specifically, it involves regressive and progressive dynamics, in the service of self-expression, self-exploration, self-understanding, and self-healing. When creativity is truly operative in art therapy, overall biopsychosocial functioning reliably improves.

Creativity requires a threshold amount of energy, and it only emerges when depression starts to lift; this is widely documented in artists with bipolar disorder symptoms. Our results show that creativity rises as energy rises, but given that we observed improvement across all scales, we cannot make causal or pathway inferences.

For all Clinical Scales, we are unable to compare low and high, or negative and positive correlations, because all Scale correlations are high and positive. Further research using multiple H-T-P assessments over time may shed light on creativity and causality, and causality generally, as will be presented in the Discussion.

One of the smallest effect sizes is found in the Cognition: Logic/Thought scale. As noted earlier, this Scale differs from the others in one notable way. The Scale is derived from the Rorschach Special Scores, which excel at detecting more severe and psychotic level cognitive impairment. Again, for our sample, psychosis was an exclusionary condition, but we made the decision to use the Rorschach Special Scores so that the Scale could be used with any PD population including persons with advanced cognitive impairments. The decision will benefit future research.

We did not obtain significant correlations with our medical team’s neuropsychological and imaging results. This will be addressed in the Discussion below.

### Case examples

Two participants will be presented here to illustrate changes in pre- and post- treatment drawings. Both participants identified as white, college-educated women with right-hand dominance. They are named here via their experimentally coded letters, participants “L” and “A.”

#### Participant L

**FIGURE 1:**
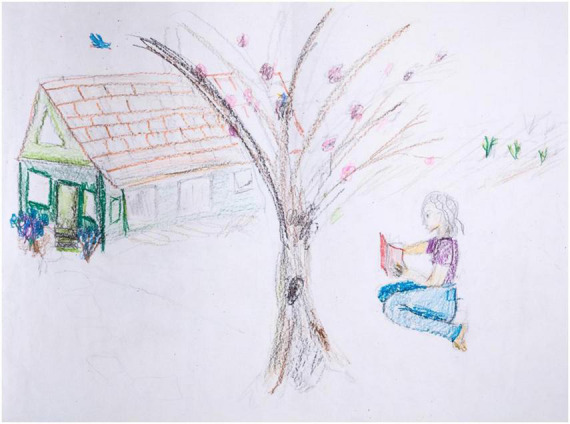
House-Tree-Person pre-treatment drawing by Participant L.

Participant L is a 68-year-old who was diagnosed with PD 10 years prior to treatment. The pre-treatment H-T-P drawing (Figure 1) depicts a large tree in the center of the paper, a woman sitting and reading a book on the right side, and a house with a slate shingle roof on the left side of the paper.

Her execution of the image reflects aesthetic inconsistencies, many of which seem PD driven, particularly line quality and visuospatial aspects. The left side of the house and tree are drawn with markedly heavy pressure, compared to the faint rectangular side of the house, wherein the lines fade into faint, short, choppy, grayed strokes. This goes beyond shading intended to indicate sunlit and shadowed areas. Nor is it an indicator of relative distance from the viewer, per atmospheric or aerial perspective, wherein nearer objects appear more saturated and brighter. Indeed, the least bright and saturated pictorial area is the nearly invisible path of flat stones that connects the front door to the foreground tree.

There are some motoric issues with the house’s front façade, in the pressured reworking, but this is predominantly due to visuospatial confusion. The front façade entails inconsistent spatial projections of the windows and door, and they are markedly misaligned with the enclosing rectangle, especially the roof line. The groundwork—the steps and dual flower beds—exacerbates the inconsistency. Apparently, she struggled with two conflicting orientations for the door, one facing us (given the stone path leading toward us) and one accurately aligned with the angled house. Similarly, abutting the facade, an additional, smaller green window is added, neither decisively on the front nor on the side of the house. Given its geometric surround—where the two walls and the roof meet—the stray window succumbs to a Necker Cube effect, with the geometry popping back and forth between competing positions. Conversely, her geometric projections are elsewhere unimpaired, especially in the triangular top half of the façade, and the parallelogram that defines the roof, even though it is incomplete in places. As a further visuospatial inconsistency, the fainter lower area of the house nearly disappears, suggesting selective inattentiveness and/or confusion regarding part/whole organization. Similarly, there is foreground/background confusion in the challenging area where the house and the tree overlap. The dark foreground tree branch divides the background roof into two distinct areas, one saturated and detailed, one abruptly blank and empty. The tree shows abandoned gray branch outlines never filled in, mixed with erasure marks—pentimenti of illogical branches that arch over the roof—both of which indicate the artist’s insight into error and motivation to self-correct.

The female figure, conversely, is well integrated (body parts), well-articulated (fine details), and thoroughly differentiated (separate from the environment). This is congruent with her emotions—note the figure’s relaxed pose and clear contentment in solitude with her book. The only problem with the depicted figure is again visuospatial. She appears to be floating upward, likely to convey relative distance by moving her closer to the implied horizon line above. But that depth cue is negated by the cue of familiar size, which indicates that she is in front of the tree (if she were behind the tree, she would be several times human size, or the tree would be miniscule). The remaining pictorial elements convey positive mood, unimpaired logic, and ample energy, such as the bluebird and the greenery. Notably, the house, tree, and person are well organized in relation to the boundaries of the page. Visuospatial aspects, like motor control, are thus part impaired, part intact, in markedly inconsistent fashion.

None of the observations regarding PD manifestations detract from the beauty of the drawing. The stated confusions, from an aesthetic perspective, introduce a challenging, at times delightful, but always interesting aesthetic ambiguity, wherein dual meanings or viewpoints are condensed in a single percept. The drawing furthermore contains enchanting visual metaphors above and beyond visuospatial confusion. As an untold example, the foreground tree branch continues directly into the background bluebird, participating in its exhilarating flight, in a poignant animistic connectedness. Because foreground and background are independent yet united, this is a controlled and articulated dual meaning. As always, the art of a patient produced in art therapy exists in two domains simultaneously—in the domain of aesthetics and in the domain of therapy.

**FIGURE 2:**
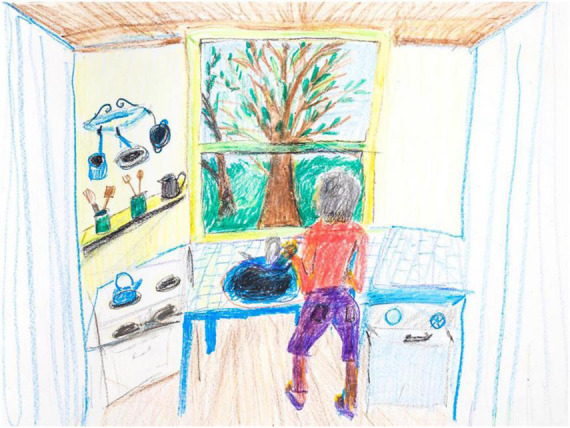
House-Tree-Person post-treatment drawing by Participant L.

Participant L’s post-drawing is Figure 2. This image features unimpaired and internally consistent pictorial indicators. Line quality varies, but intentionally and successfully, and in the service of depicting illumination gradations (shaded to bright areas), and texture gradients (near to far areas). Foreground and background are decisively delineated, with the trees accurately occluded by the foreground window frame, itself steady and geometric. The geometry is decisive and fairly accurate throughout the room, by dint of converging lines that serve perspective, albeit with some persistence of the Necker cube effect. Generally, items are solid, especially in the shelf area. The kitchenware is well detailed and articulated, evidencing increased motor control and confidence. The mood is no longer mixed, it is positive. Instead of a barren house, she has moved in, busily washing dishes in the overabundant kitchen, itself a nurturing setting. She is now well grounded. The tree(s) is now solid and enlivened. Energy and color are robust. The drawing fills the entire page. The improvement over the pre-treatment drawing goes well beyond aesthetic sophistication pursuant to drawing practice; rather, PD core symptoms appear ameliorated, along with a general enhancement of mood, motivation, and sense of self.

Participant L’s pre- and post-treatment scores on the HTP-PDS are exhibited in Table 2. All variables improve except Cognition where there is no baseline impairment. The clinical and behavioral results are found in Table 3.

**TABLE 2:** HTP-PDS results for Participant L.

	Pre-intervention HTP-PDS	Post-intervention HTP-PDS	Difference scores
Motor control	4	5	+1.0
Visual/spatial functioning	3	5	+1.0
Cognition: logic/thought	5	5	0.0
Motivation	4	5	+1
Emotion	3.5	5	+1.5
Self	3	4	+1
Interpersonal	3	4	+1
Creativity	4	5	+2.0
Global assessment of functioning	3	5	+2.0

**TABLE 3:** Clinical and behavioral results for Participant L.

Measurement	Baseline	Follow up
UPDRS_III_	31	26
NAVON_TotalErrors_	1	1
BECK depression inventory	10	5

#### Participant A

**FIGURE 3:**
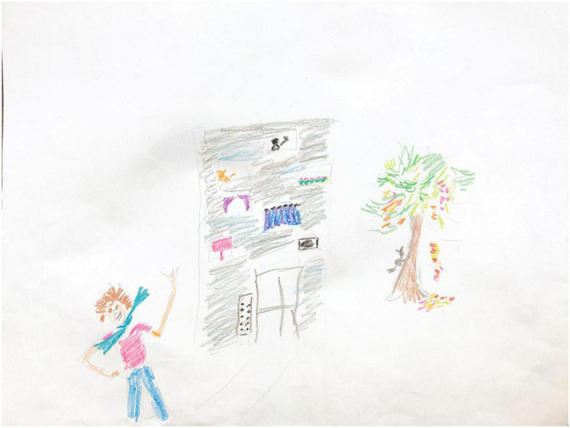
House-Tree-Person Pre-treatment drawing by Participant A.

Participant A is a 58 year old woman diagnosed with PD 17 years ago. In this pre-treatment drawing (Figure 3), the artist begins with faint gray outlines and fills them in with color afterward, drawn with short and choppy strokes. Elsewhere, back and forth scribbles appear, executed without lifting the crayon from the page, perhaps for ease of execution, perhaps to avoid errors that are more likely with independent strokes. Fine line placement, too, is inconsistent in the house’s door and windows, some of which are intact, suggesting ability that is compromised by low motivation or impatience. Still, the artist succeeds with this strategy in the brilliant tree foliage, where choppy strokes produce vibrant beauty, variegated color, and animated motion, as seen in the falling leaves. The house’s façade is also vibrantly variegated and colored, but with the opposite effect: the windows are quirky and oddly colored, suggesting some mild illogic. The chief problem here is visuospatial organization, given the random placement of the windows, and given that the house itself is flat. The flat facade suggests a strategy to keep things simple; similarly the house, tree, and person don’t interact or overlap, foreclosing confusion.

The human figure appears joyful but displays a bold gesture rather than genuine emotion. The figure is turned away from the house and the tree, seemingly avoidant of her own created environment. Notably, the feet are cropped, implying immobility, and the figure is positioned at a marked angle, implying instability. She is drawn with sharp angles and appears flattened and stiff.

**FIGURE 4:**
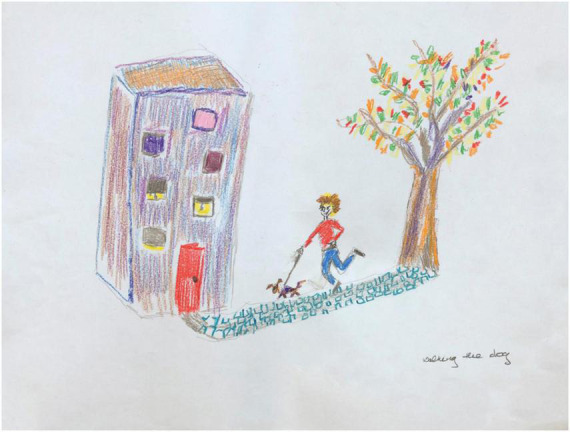
House-Tree-Person post-treatment drawing by Participant A.

The post-treatment drawing (Figure 4) has substantially evolved. Smooth and continuous lines define the house outline. The windows, door, and walkway exhibit controlled fine line placement. The artist now revels in the ability to execute multiple types of lines to suit the depicted content, as evident in the choice to depict a challenging cobblestone path. Visuospatial aspects have improved too; the house is now three dimensional, and the windows, while still staggered, appear playful rather than odd. The person is integrated with the house and the tree, connected by a path. The elements are all in balance with each other and with the paper borders, evidence of markedly improved spatial organization. The person is no longer immobilized, angular, unstable, and isolated, and now walks confidently, accompanied by a lively pet. Energy and color abound; mood, sense of self, and environmental relatedness have all improved.

Participant A’s pre- and post-treatment scores on the HTP-PDS are exhibited in Table 4. The clinical and behavioral results are found in Table 5.

**TABLE 4:** HTP-PDS results for Participant A.

	Pre-intervention HTP-PDS	Post-intervention HTP-PDS	Difference scores
Motor control	2.5	4.00	+1.5
Visual/spatial functioning	2.5	4.0	+1.5
Cognition: logic/thought	4.0	4.5	+0.5
Motivation	2.5	4.5	+2.0
Emotion	3.5	4.5	+1.0
Self	2.5	4.5	+2.0
Interpersonal	3	4.5	+1.5
Creativity	2.5	4.5	+2.0
Global assessment of functioning	2.5	4.5	+2.0

**TABLE 5:** Clinical and behavioral results for Participant A.

Measurement	Baseline	Follow up
UPDRS_III	25	11
NAVON_Total Errors_	5	4
BECK DI	3	0

**FIGURE 5:**
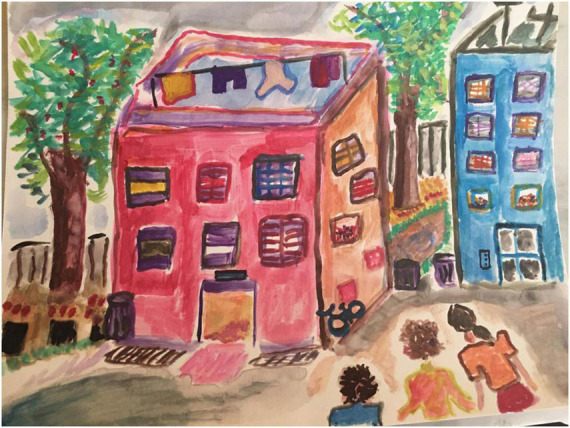
House-Tree-Person drawing by Participant A: 1 year after treatment (remote sessions).

The research project was completed in 2019, however, in 2020, due to the Covid pandemic, the art therapy team offered open community-based art therapy. Participant A voluntarily participated via Zoom sessions using a desktop computer. At our suggestion, she did another HTP drawing; this permits some inferences about the long-term effects of the art intervention. Figure 5 is her HTP drawing. She chose watercolor, her stated favorite medium. The work evidences confidence and success in motor control throughout. Visuospatial aspects have also progressed; this scene is much more complex, and takes more risks, and shows mastery of multiple depth cues. Receding orthogonal lines converge; the trees and house decisively occlude the background fence. All pictorial elements are integrated and balanced. The page is now filled to the edges. Mood, sense of self, motivation, and interpersonal engagement are all entirely positive. The human figure is no longer chopped by the picture frame, rather she has acquired two friends, and they enter the picture confidently, and with sophisticated, lifelike poses. Participant A identified one of three figures to be herself, standing alongside friends, holding hands, appreciating all of New York City.

## Discussion

While improvements were found across all scale categories of the HTP-PDS, the possible confounds of talent, practice, and maturation between pre- and post-assessments are to be considered. Generally, projective tests that tap into organicity as well as personality variables are immune to these confounds. Still, all creative art products are multi-determined by factors including talent, practice, maturation, aesthetic sensitivity, and so forth, which evolve synergistically over time. Artistic evolution is variously manifest in art students, in the art of developing children, and in cultural progressions throughout art history. Further research on the HTP-PDS is needed to disentangle the foregoing variables from what we are attempting to study, namely organic PD manifestations and projective personality aspects. In future research it will be prudent to give participants a drawing skills test (for a baseline measure), and furthermore, to include a drawing class control condition (to disentangle arts practice from art therapy). Further control conditions will be noted in context.

In the present study, pre- and post-drawings were scored in pairs, together, by blind raters. The two drawings beheld simultaneously provide necessary context for interpretation, as in clinical practice. It is possible that viewing paired drawings pulls for exaggerated difference scores, not to mention a halo effect (wherein high rating on one variable pulls for high ratings on other variables for a given drawing). Still, improvements are plainly visible in most drawing pairs, and no pairs evidence obvious deterioration. A related limitation is the raters’ knowledge of the research hypotheses, although trained, double blind raters achieved similar results on a sample of drawings.

An inherent limitation is the relative novelty of the HTP-PDS. While sufficiently reliable and valid for our immediate purposes, it remains a work in progress, with psychometric properties and construct validity expected to mature with future research and further refinements. Naturally so—it is a daunting task to quantify free drawings. The benefits of a holistic task are matched by the inherent difficulties of establishing reliability and validity. This holds true for all arts-based, projective assessment instruments.

We expected that changes in core symptoms—motoric and visuospatial processing—would beget global personality improvements. The experimental design, however, did not disentangle correlation from causation, partly due to consistently high correlations among all variables. Future research will include multiple assessments across the treatment period to facilitate causal inferences (i.e., does improvement in motor and visuospatial at times A or B predict improvements in personality globally at points C or D, more so than the other way around?).

Additionally, due to logistics, we did not include any control conditions, such as PD patients on medication, or PD patients undergoing other complementary therapies. In addition to the control condition suggested above—studio arts classes without the therapy component of art therapy—we are strategizing ways to isolate and examine each of the diverse healing principles hypothesized to underlie art therapy.

To explain the strategy, a quick recap of some of the healing principles will be useful. Many hypotheses have been formulated regarding the mechanisms through which art therapy effects therapeutic change (Berberian, 2020; de Witte et al., 2021; Galassi et al., 2022). Artmaking is believed to involve a state of intense absorption, or “Flow” (Csikszentmihalyi, 1991), potentially resulting in an analgesic escape from PD symptoms and related cumulative disability. This process may indeed hold therapeutic potential in mitigating the functional impact of chronic symptoms which would otherwise amplify and perpetuate due to the progressive, unremitting nature of the underlying neurodegenerative disorder.

Visual artmaking furthermore involves the expression, in non-verbal form, of deep and multilayered psychological phenomena, especially those stored in visual and somatic codes. Once these are safely externalized in a tangible medium, they can be faced, explored, understood, integrated, and reorganized through verbal dialogue, effecting psychological relief and change. Artistry furthermore involves total symbolic control over this self-generated, internal world, promoting self-efficacy in everyday life. This is especially apropos for PD patients, insofar as art-making entails learning new sensorimotor skills which could be relatively spared by the neurodegenerative process of PD, as the latter is known to mainly affect the planning and execution of semiautomated, internally generated motor tasks.

Here are two examples of control conditions suggested by the foregoing healing principles. If personalized, symbolic expression is a curative factor, we can instruct a control group to trace or copy extant artworks, selectively removing personal creativity from the mix. If absorption and Flow are curative factors, akin to mindful meditation, we can design an art therapy protocol involving heightened external interaction and distraction, selectively removing the introspective conditions that lead to absorption.

We did not include a PD placebo intervention. This was a decision made with ethics in mind—we did not want to deprive patients of available, validated therapies. As a general rule, a placebo condition with structure, safety, care, empathy, and interpersonal relatedness will yield some positive results. These same factors are operative in art therapy, and it remains a philosophical question as to whether they should be considered extraneous or inherent treatment factors. In one eminent view of complementary treatments “about 15–30% of the treatment effect is based on factors that are shared with a good placebo condition” (Ed Taub, personal communication, February 6, 2017, T.E.).

Future research using the HTP-PDS will include control conditions to determine whether art therapy is protective against deterioration in PD populations. Some recent research citing our earlier publications suggests that art therapy is prophylactic in this regard. Khubetova and Vorokhta (2021) assigned 50 PD patients to a medication condition or a medication plus art therapy condition; after 1 year of treatment, 33% of the medication alone patients deteriorated compared to 10% of the medication and art therapy patients (deterioration was operationally defined as a.5 decline on the Hoehn and Yahr Scale). It is therefore possible that our positive pre/post-differences are superimposed upon an undetected functional decline. If so, then any detected improvements, significant or not, are clinically meaningful. We will assess this possibility in future research by measuring baseline HTP-PDS decline with proper control conditions, including medication alone.

Finally, we did not find significant correlations with our neurological tests and imaging studies as reported by Cucca et al. (2018, 2021). In arts-based research this is a common finding and a lingering quandary. For example, Khubetova and Vorokhta (2021) obtained positive results on personality and wellness questionnaires consistent with their deterioration/improvement indices. However, they did not find significant UPDRS results; only Pegboard test results were significant. The Pegboard test measures eye/hand coordination and fine musculature dexterity. This closely mirrors what is learned and practiced in art therapy. More generally, art therapy is likely to involve peculiar functional domains mediated by complex neural substrates which are not necessarily reflected in traditional clinical and neuropsychological outcomes. This is especially likely for PD patients, insofar as the acquired and evolving sensorimotor skills are relatively spared by the neurodegenerative process of PD, as the latter is known to mainly affect the planning and execution of semiautomated, internally generated motor tasks. An important goal in neuroscience research is the development of dependent measures that more accurately assess the biopsychosocial benefits of arts-based therapies.

## Conclusion

The HTP-PDS results, with all caveats considered, offer further evidence to a burgeoning literature that supports the use of art therapy as a complementary intervention. Art therapy treatment is an effective rehabilitative tool with robust biopsychosocial benefits and no side effects. More generally, multidisciplinary approaches involving complementary therapies have tremendous potential to augment traditional medication and physiotherapies, for PD as well as other organic and degenerative diseases. Future research in this area will benefit from isolating and assessing the hypothesized healing mechanisms of arts-based complementary therapies, for the purposes of optimizing treatment protocols. Additionally, the development of domain specific assessment instruments holds promise for all arts-based therapies. When the assessment modality matches the treatment modality, sensitivity to positive change is optimized—albeit there are pernicious confounds that must be overcome.

## Data availability statement

The original contributions presented in this study are included in the article/supplementary material, further inquiries can be directed to the corresponding author.

## Ethics statement

The studies involving human participants were reviewed and approved by NYU Langone Health. The patients/participants provided their written informed consent to participate in this study.

## Author contributions

TE: primary author and editor. MgB, AC, JR, and IA: contributors. TE, MgB, and IA: art therapy researchers: scale development [HTP-PDS]. MgB, IA, and RK: clinicians. RK and CG: research assistants. AC, JR, DG, and MB: Parkinson’s disease medical researchers. AF, AD, FG, and J-RR: senior research supervisors. AC, MB, GK, TP, and TE: advisors and statisticians.

## Footnotes

1 The HTP-PDS Manual will be made available to qualified researchers who wish to replicate or extend or otherwise adapt it for new clinical populations. Please contact Dr. Ettinger for this.
